# Elevated C-Reactive Protein Levels Modify the Effect of Magnesium on Depressive Symptoms: A Population-Based Study

**DOI:** 10.3390/nu15071560

**Published:** 2023-03-23

**Authors:** Ming-Hui Chou, Yen Kuang Yang, Jung-Der Wang, Chung-Ying Lin, Sheng-Hsiang Lin

**Affiliations:** 1 Department of Public Health, College of Medicine, National Cheng Kung University, Tainan 704, Taiwan; 2 Department of Psychiatry, National Cheng Kung University Hospital, College of Medicine, National Cheng Kung University, Tainan 704, Taiwan; 3 Institute of Behavioral Medicine, College of Medicine, National Cheng Kung University, Tainan 704, Taiwan; 4 Department of Psychiatry, Tainan Hospital, Ministry of Health and Welfare, Tainan 700, Taiwan; 5 Department of Occupational and Environmental Medicine, National Cheng Kung University Hospital, College of Medicine, National Cheng Kung University, Tainan 704, Taiwan; 6 Institute of Allied Health Sciences, College of Medicine, National Cheng Kung University, Tainan 704, Taiwan; 7 Biostatistics Consulting Center, National Cheng Kung University Hospital, College of Medicine, National Cheng Kung University, Tainan 704, Taiwan; 8 Institute of Clinical Medicine, College of Medicine, National Cheng Kung University, Tainan 704, Taiwan

**Keywords:** serum magnesium, depression, depressive symptoms, C-reactive protein (CRP), dose–response, Nutrition and Health Survey in Taiwan (NAHSIT), inflammation

## Abstract

Depression is a profound public health concern, yet its etiology remains unclear. A body’s magnesium status and low-grade systemic inflammation are associated with depression. However, the interaction of magnesium status and inflammation on depression/depressive symptoms is unknown. We assessed the association between serum magnesium levels and depressive symptoms by analyzing data from the Nutrition and Health Survey in Taiwan 2005–2008. In total, 2196 participants aged ≥20 years were included. Depressive symptoms were assessed using the 5-item Brief-Symptom Rating Scale. We performed logistic regression and multiple linear regression analyses to examine the association. A dose–response analysis was performed using restricted cubic spline models, and stratification by chronic inflammation was also performed. We found that higher serum magnesium levels were associated with lower depression scores and a lower risk of depression. In the subgroup analysis, serum magnesium levels were inversely associated with depressive symptoms more prominently among people with higher CRP levels, with a threshold at 5 mg/L (≥5 vs. <5) showing a greater difference than at 3 mg/L (≥3 vs. <3). Conclusions: Serum magnesium levels were inversely associated with depressive symptoms. This inverse association was affected by inflammation level. A dose–response relationship was also observed.

## 1. Introduction

Depression is among the leading causes of the global burden of disease and the top cause of disability measured on the basis of years lived with disability (YLD) [1,2]. The pathogenesis of depression is not completely understood. The heterogeneity of depression presents in symptoms, possible etiologies, and comorbidities, such as chronic diseases and psychiatric disorders. A lot of hypotheses have been proposed, but no one has been able to fully recapitulate the entire human depression syndrome [3]. It is estimated that one-third of depressive patients do not respond to antidepressant treatment [4]. The low response rate reflects the fact that current knowledge of depression needs further improvement. Health concerns regarding the effects of nutrition on depression, especially minerals such as magnesium, have been increasing in the recent decade [5,6,7]. Magnesium is the second most abundant intracellular cation in humans, and it participates in more than 600 enzymatic reactions, including those involved in energy metabolism and protein synthesis [8]. An estimated 45% of Americans are magnesium deficient and are thus prone to numerous diseases, such as hypertension, diabetes, and neurological disorders [9,10]. A similar condition was also observed in Taiwan; the prevalence of low serum magnesium levels (<0.8 mmol/L) was 12.3% and 23.7% for males and females, respectively [11].

The exact pathophysiology of depression and role of magnesium are unclear, but nonetheless could be based on several pathways. Magnesium affects the response of the hypothalamic–pituitary–adrenal (HPA) axis. Studies have shown that magnesium deficiency causes the release of corticotropin-releasing hormones in the paraventricular hypothalamic nucleus, which increases the level of adrenocorticotropic hormone (ACTH) and causes hyperarousal of the HPA axis [12,13]. HPA excitability elevates the levels of stress hormones associated with depression, including norepinephrine, dopamine, and cortisol [14]. Magnesium also regulates glutamate signaling and is a *N*-methyl-d-aspartate (NMDA) receptor antagonist. NMDA receptors are activated by the binding of glutamate and return to their resting state with the binding of magnesium. Magnesium deficiency causes NMDA-coupled calcium channels to reach an activated state, which leads to neuronal damage [15]. Additionally, the dysfunction of NMDA receptors plays a crucial role in the development of depression [16,17]. Studies have indicated an association between dietary magnesium intake and depression risk in the community-dwelling population of the United States [18,19], and Sun et al. further demonstrated a dose–response effect. Dose–response analyses have been widely applied in studies investigating the association between magnesium intake/serum magnesium and diseases, including depression [20,21,22,23]. Nevertheless, because of the complicated biological mechanism of absorption and the sensitive compartmental handling of magnesium, dietary magnesium intake is not necessarily indicative of a body’s magnesium status [9]. Accordingly, studies assessing the association between magnesium status and depression have widely adopted serum magnesium as an indicator of magnesium status [7,24,25,26,27].

Magnesium deficiency was also associated with low-grade inflammation and related systemic diseases [28,29]. Inflammation may cause depression, but the underlying pathology between inflammation and depression is not completely understood. Studies have shown that cytokines might lead to the development of depressive symptoms through the activation of indoleamine-2,3-dioxygenase, which breaks down tryptophan into kynurenine, consequently decreasing the production of serotonin and increasing the production of kynurenic and quinolinic acids [30,31]. Decreased serotonin levels are crucial players in the pathophysiology of depression. Meanwhile, an increased production of quinolinic acids directly triggers glutamate release and increases the quinolinic acid to kynurenic acid ratio, leading to net NMDA agonism, which may mediate the development of depression [32]. Magnesium deficiency is associated with higher levels of C-reactive protein (CRP) and its precursors, such as interleukin (IL)-6 and IL-1, both of which are positively associated with the incidence and severity of depression [33]. CRP is a commonly used marker of chronic systemic inflammation, and elevated CRP levels have been associated with depression [34]. Although CRP itself cannot cross the blood–brain barrier, elevated CRP levels probably indicate elevated levels of cytokines, which can cross this barrier and have been proven to be associated with depression [35]. The American Heart Association defined peripheral CRP levels greater than 3 mg/L as “high CRP” and as associated with the greatest risk for cardiovascular disease, relative to CRP levels less than 1 mg/L (low CRP) and between 1 and 3 mg/L (moderate CRP) [36]. This definition was also applicable to evidence that increased CRP levels are associated with depression and elevated central nervous system biomarkers [34,35]. Nonetheless, studies focusing on the relationship between magnesium status and depression have rarely considered baseline inflammation. Previous studies have also proved baseline CRP levels to be a reliable predictor of response to anti-inflammatory agents from inflammatory diseases [37,38]. An inflammation threshold of 5 mg/L, compared to one of 3 mg/L, was also found to make a difference in agent effectivity in an intervention study [39]. If the pathogenesis of magnesium deficiency on depression were involved in inflammation, we would hypothesize that the protective effect of higher serum magnesium against depressive symptoms might be stronger in those with a higher inflammation level.

In this study, we assessed the association between serum magnesium levels and depressive symptoms in a community-dwelling population in Taiwan. We also investigated whether this association was more prominent at higher CRP levels. To control confounders, we adjusted our models for age, sex, education level, income, physical activity status, alcohol use, smoking status, diabetes mellitus, hypertension, and kidney disease.

## 2. Materials and Methods

### 2.1. Study Population

We analyzed de-identified data from the Nutrition and Health Survey in Taiwan (NAHSIT) 2005–2008. The survey included questionnaire data and physical examination results. This study collected data on 4615 non-institutionalized individuals aged ≥20 years from between 2005 and 2008. We excluded data on participants for whom any of the following information was missing: (1) 5-item Brief Symptom Rating Scale (BSRS-5) scores, (2) any of the sociodemographic information, and (3) blood biochemical parameters. Data from a total of 2196 participants were included in this study. Informed consent was obtained from all participants, and the study was approved by National Cheng Kung University’s Research Ethics Review Board (IRB #B-EX-110-014).

Depression status was treated as both a continuous variable and a binary variable and was assessed using the BSRS-5. The reliability and validity of the BSRS-5 were assessed in different populations. The internal consistency (Cronbach alpha) coefficients of the BSRS-5 ranged from 0.77 to 0.90. The test-retest reliability coefficient was 0.82. The BSRS-5 can be used to identify psychiatric morbidity in both medical practice and the community [40]. The participants were asked to report on a five-point Likert scale the extent to which feeling down and depressed had distressed or bothered them during the past week (including the current day); the scale ranged from 0 to 4 (0 = not at all; 1 = a little bit; 2 = moderately; 3 = quite a bit; and 4 = extremely) [40]. We also categorized depression scores into two groups: (1) item scores of <2 as negative and (2) item scores of ≥2 as positive for symptoms of depression.

### 2.2. Assessment of Serum Magnesium

Concentrations of serum magnesium were measured using a colorimetric assay on the Roche Cobas Integra 800. In the colorimetric method, an alkaline complex with absorption at 520 nm is formed when magnesium in the blood reacts with xylidyl blue. The formation of alkaline complex is positively associated with levels of serum magnesium. Glycol ether diamine-*N*,*N*,*N*′,*N*′-tetraacetic acid (GEDTA) was used to avoid calcium interference. Blood was drawn from subjects of NAHSIT 2005–2008 in the morning.

### 2.3. Assessment of C-Reactive Protein

CRP concentrations were obtained from blood samples in the 2005–2008 Nutrition and Health Survey in Taiwan (NAHSIT) 2005–2008. CRP concentrations were measured using a particle-enhanced immunoturbidimetric test with an automatic analyzer (Hitachi 747; Hitachi, Tokyo, Japan). The limit of detection was 0.07 mg/dL.

### 2.4. Other Covariates

Potential depression-related confounding variables were examined, including sociodemographic information, lifestyle, and medical history. Age was considered a continuous variable (range: 20–101 years). Based on their reported sex, participants were categorized as women or men. Body mass index (BMI) was divided into three groups: <18, 18–24, >24. Education levels were divided into four groups: none, below senior in high school (SHS), SHS, and above SHS. Monthly income levels were classified into five groups: no income, TWD 20,000 and below, TWD 20,001–50,000, TWD 50,001–70,000, and TWD 70,001 and above. Physical activity status was grouped based on three responses: (1) no, (2) yes–only walking, and (3) yes–more than walking. Physical activities considered as “more than walking” included running, hiking, folk dancing, aerobic dancing, swimming, and bicycling. Smoking status and alcohol use were each categorized into three groups: non-smoker, former smoker, and current smoker; and non-drinker, former drinker, and current drinker. A participant’s history of comorbidities (e.g., hypertension, kidney disease, and diabetes mellitus) was considered a dichotomous variable (yes vs. no). Serum CRP levels were grouped into two clinically relevant categories: (1) <3 mg/L and (2) ≥3 mg/L.

### 2.5. Statistical Analysis

We performed a chi-square test and Fisher’s exact test for categorical variables, and a t-test or an ANOVA test for continuous variables. No multicollinearity was observed in the adjusted models using variance inflation factors. We performed multiple linear regression analyses when depression score was treated as a continuous variable and a built logistic regression model when depression score was treated as a binary variable. We reported a crude association between depression and serum magnesium levels and an association between depression and CRP levels. Model 1 was adjusted for age and sex, and Model 2 was adjusted for statistically significant variables (*p* < 0.05) in the univariate analysis. Model 2 was additionally adjusted for BMI and physical activity status when depression score was treated as a continuous variable. When depression score was treated as a binary variable, Model 2 was adjusted for age, BMI, income, physical activity status, smoking status, and kidney disease. Subgroup analyses by CRP level and sex were also performed. Restricted cubic spline model functions were used to assess the dose–response relationship between depression, as a binary variable, and serum magnesium levels using the %LGTPHCURV9 macro in the SAS software [41]. All analyses were conducted using SAS version 9.4 (SAS Institute, Cary, NC, USA).

## 3. Results

The characteristics of the included subjects are listed in Table S1. The participants chosen for this study had significantly higher education levels and incomes, were more physically active, and were less likely to be current smokers than the average survey respondent. 99.45% of their blood samples were collected in the morning so diurnal variation could be minimized. Table 1 presents the characteristics of predictors by depression score as a continuous or binary variable. Most participants had below SHS education levels (936/2196; 42.62%) and earned TWD 20,000 or below per month (842/2196; 38.34%). They were non-drinkers (1186/2196; 54.01%), non-smokers (1493/2196; 67.99%), physically inactive (978/2196; 44.54%), had BMIs higher than 24 (1145/2196; 52.14%), and had CRP levels < 5 mg/L (1976; 89.98%). In addition, most participants did not have diabetes mellitus (2007/2196; 91.39%), hypertension (1665/2196; 75.82%), or kidney disease (2156/2196; 98.18%). Participants’ sociodemographic characteristics differed significantly for age, income, smoking status, physical activity status, kidney disease, and CRP levels when depression status was treated as a binary variable. People who had depression scores of <2 were older, more physically active, more likely to be non-smokers, and had lower CRP levels and higher serum magnesium levels than those with a depression score of ≥2. When depression was treated as a continuous variable, the mean depression score was significantly higher among women, younger participants, people who were not physically active, and those with lower serum magnesium levels. 

Table 2 presents the results of the logistic regression and multiple linear regression analyses, where serum magnesium and CRP levels were used as key independent variables. Model 1 was adjusted for age and sex, and Model 2 was adjusted for statistically significant variables in the univariate analysis. When depression score was treated as a continuous variable, serum magnesium levels were significantly inversely associated with depression scores (β = −0.23; 95% CI = −0.38, −0.07; *p* = 0.004). Individuals with CRP levels ≥ 5 mg/L (compared to those with CRP levels < 5 mg/L) were significantly associated with higher depression scores (β = 0.14; 95% CI = 0.04, 0.24; *p* = 0.005). When depression score was treated as a binary variable, serum magnesium levels were statistically negatively associated with depression scores (OR = 0.37; 95% CI = 0.16, 0.87; *p* = 0.022), and individuals with CRP levels ≥ 5 mg/L (compared to those with CRP levels < 5 mg/L) had odds ratios (ORs) of depression greater than 1 (OR = 2.19; 95% CI = 1.35, 3.55; *p* = 0.002). These results, categorized by CRP levels ≥ 3 mg/L, are shown in Table S2.

**Figure 1:**
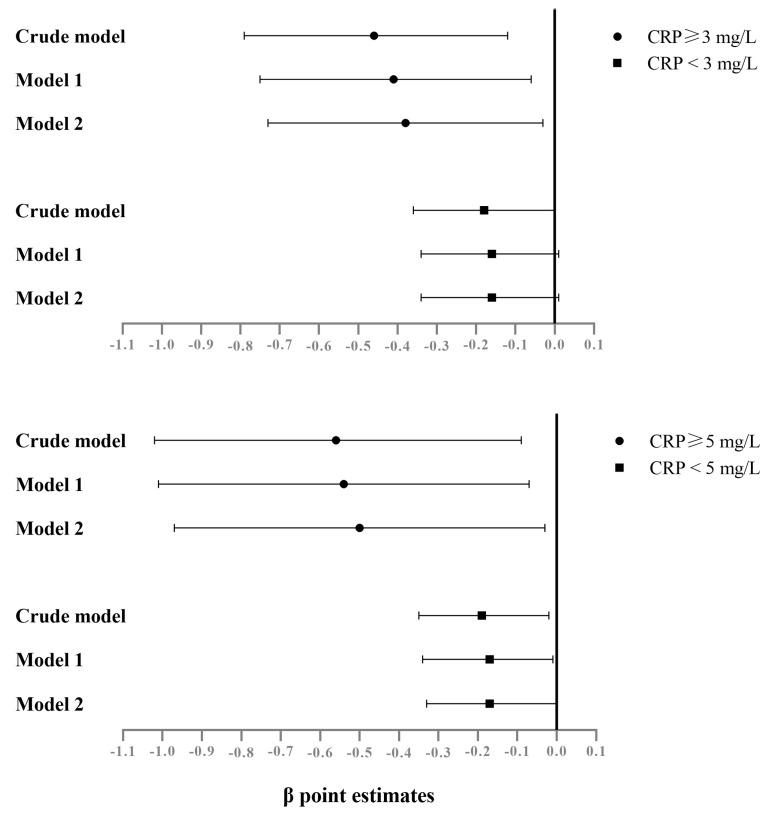
Forest plot for β (regression coefficients) of depression with serum Mg levels, categorized by different CRP levels, NAHSIT 2005–2008. The error bars indicate a 95% CI. Model 1 was adjusted for age and sex. Model 2 was adjusted for statistically significant variables (*p* < 0.05) in the univariate analysis (Table 1).

**Figure 2:**
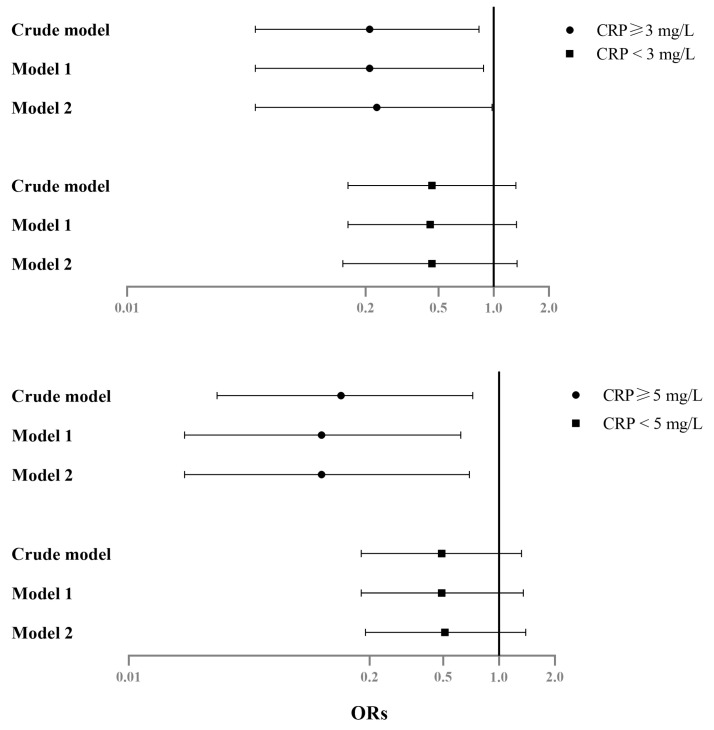
Forest plot for odds ratios (ORs) of depression with serum Mg levels, categorized by different CRP levels, NAHSIT 2005–2008. The error bars indicate a 95% CI. Model 1 was adjusted for age and sex. Model 2 was adjusted for statistically significant variables (*p* < 0.05) in the univariate analysis (Table 1).

Table 3 presents the results of the logistic regression and multiple linear regression analyses stratified by CRP levels. In the linear analysis, serum magnesium levels were inversely associated with depression scores in the groups of CRP levels ≥ 5 mg/L (β = −0.50, 95% CI = −0.97, −0.03; *p* = 0.039) and of <5 mg/L (β = −0.17, 95% CI = −0.33, −0.00; *p* = 0.045). In the logistic regression analysis, serum magnesium levels were related to lower ORs of depression in those with CRP levels ≥ 5 mg/L (OR = 0.11; 95% CI = 0.02, 0.69; *p* = 0.018), but its association to lower ORs of depression was not statistically significant in those with CRP levels < 5 mg/L (OR = 0.51; 95% CI = 0.19, 1.39; *p* = 0.186). These results, categorized by CRP levels ≥ 3 mg/L, are shown in Table S3. The β (regression coefficients) of linear regression and ORs of logistic regression are also illustrated in Figure 1 and Figure 2, respectively.

Tables S4 and S5 present the results of the gender-stratified analysis. Serum magnesium levels were significantly inversely associated with depression scores among men, whereas this association was insignificant among women.

**Figure 3:**
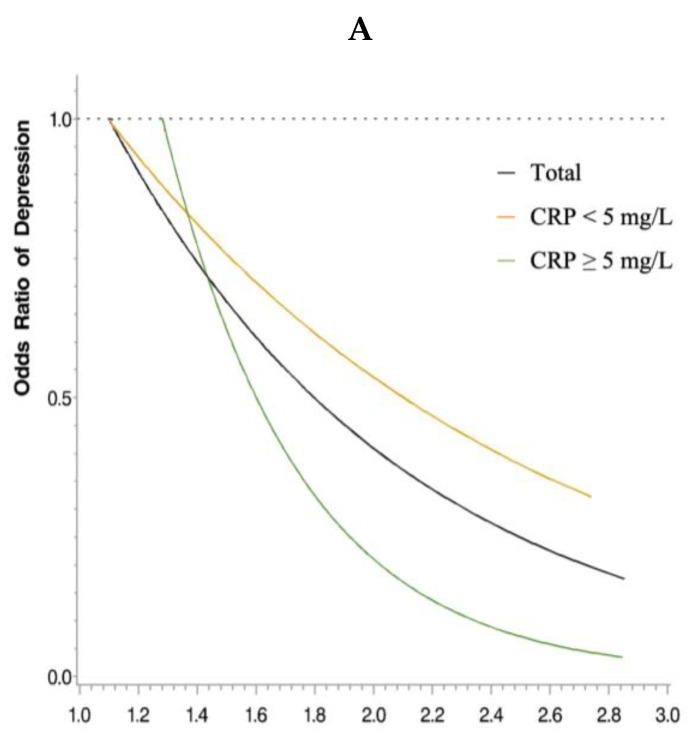

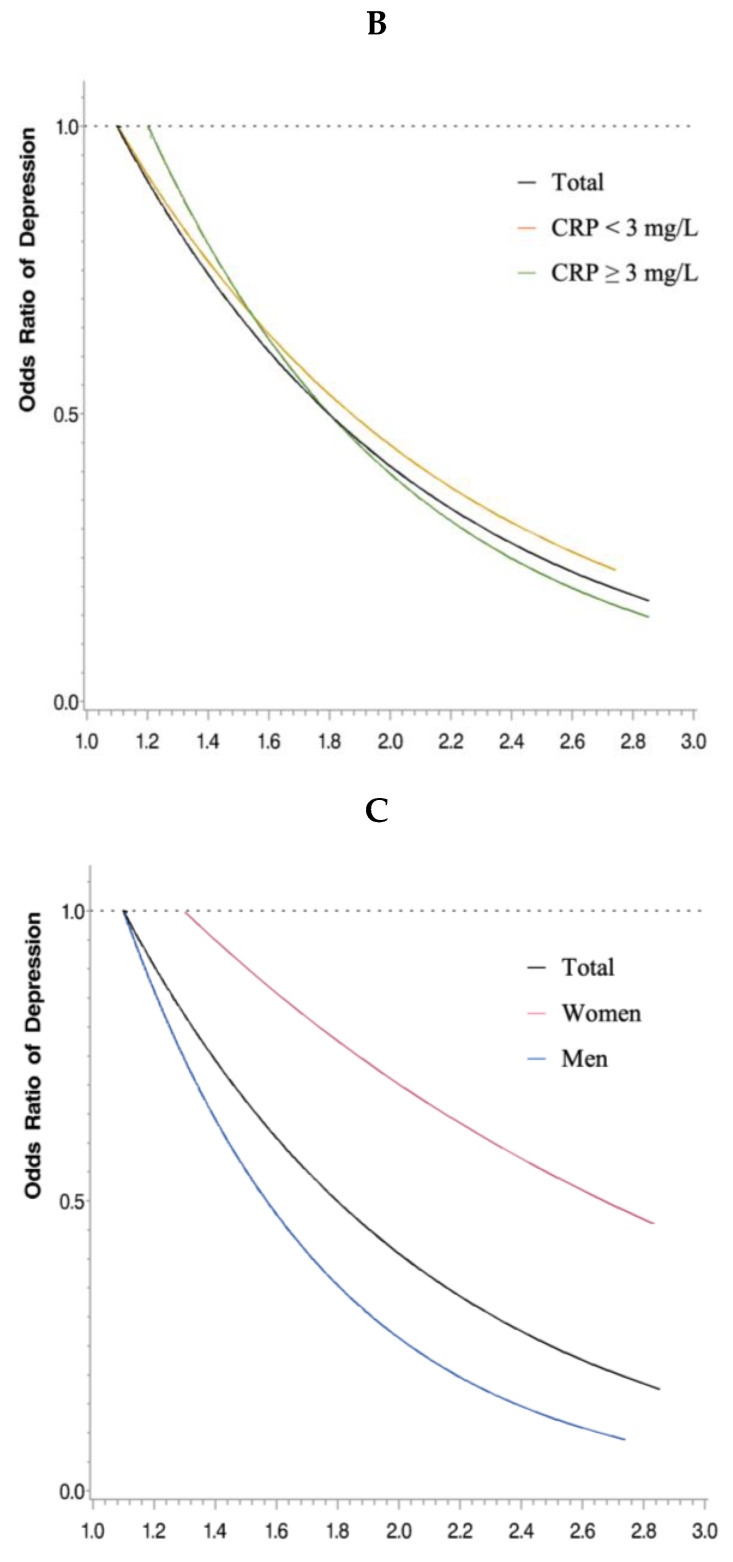
The restricted cubic spline model of the odds ratios of depression with serum magnesium levels (mg/dL). (**A**) The black line represents the total sample, the orange line represents CRP levels < 5 mg/L, and the green line represents CRP levels ≥ 5 mg/L. (**B**) The black line represents the total sample, the orange line represents CRP levels < 3 mg/L, and the green line represents CRP levels ≥ 3 mg/L. (**C**) The black line represents the total sample, the pink line represents women, and the blue line represents men. All models were adjusted for age, income, smoking status, physical activity status, kidney disease, and CRP levels.

The dose–response relationship between serum magnesium and depression risk was examined using restricted cubic spline analyses. Figure 3 shows the linear relationship (P for nonlinearity: overall = 0.318, CRP ≥ 5 mg/L = 0.007, CRP < 5 mg/L = 0.753, men = 0.381, women = 0.214) between serum magnesium and depression, which was statistically significant in the overall sample, in subjects with CRP levels ≥ 5 mg/L, and in men (*p* value: overall = 0.030, CRP ≥ 5 mg/L = 0.039, men = 0.016, respectively).

## 4. Discussion

After adjusting for potential confounders, applying two different statistical models, and conducting a stratification analysis, we consistently found serum magnesium levels to be inversely associated with depression scores, especially among people with elevated CRP levels. To our knowledge, this study is the first to demonstrate the association between serum magnesium and depressive symptoms in community-dwelling populations while taking inflammation into consideration. Serum magnesium levels were inversely associated with depression scores more prominently among people with higher CRP levels, with a threshold at 5 mg/L (≥5 vs. <5) showing a greater difference than at 3 mg/L (≥3 vs. <3). Serum magnesium levels were negatively associated with depression scores in the overall sample among men, but not among women. 

Similar to a previous study, our results showed that serum magnesium levels were negatively associated with depression scores [7]. A similar conclusion has also been drawn in dietary magnesium studies [19,42]. Sun et al. reported that dietary magnesium intake was negatively associated with depression, which was congruent with our results. However, they found that dietary magnesium intake was negatively associated with depression among women, but not among men, whereas in our study, serum magnesium levels were negatively associated with depression scores among men, but not among women. This discrepancy might have resulted from differences in methodology. Sun et al. measured dietary magnesium intake, whereas we used serum magnesium to evaluate magnesium status in the present study. Because magnesium metabolism is unique, dietary magnesium is not necessarily representative of the functional status of magnesium within the body. In addition, the evaluation of dietary magnesium intake in the study performed by Sun et al. was possibly affected by factors such as recall bias and the variation of nutritional components in the same food. Declining rates of food magnesium have been reported due to food processing and agronomic factors [43,44]. Thus, serum magnesium might reflect the functional status of magnesium more directly and correlate with depression better. These factors could probably explain the differences between these two studies.

Sex hormones could also possibly explain why our results showed an association only in men and not in women. The cyclic fluctuation of estrogen and progesterone increases women’s vulnerability to depressive symptoms and affects the presentation of these symptoms [45]. In addition, serum magnesium levels are altered by sex hormones during different phases of the menstrual cycle [46], which would impact the readings of serum magnesium. Together, these factors diminish the association between serum magnesium levels and depressive symptoms in women.

Magnesium deficiency contributes to the development of depressive symptoms through inflammation, HPA axis hyperactivity, neurotransmitter dysregulation, and NMDA receptor dysfunction. The protective effect of serum magnesium against depressive symptoms was more prominent in individuals with higher inflammatory levels. When treating depression as a binary outcome, serum magnesium was associated with ORs of depression in individuals with CRP levels ≥ 5 mg/L, but not in those with CRP levels < 5 mg/L. For each 1 mg/dL increase in serum magnesium, the depressive score decreased by 0.50 for those with CRP levels ≥ 5 mg/L, but only by 0.16 for those with CRP levels < 5 mg/L. Therefore, CRP levels affected the association between serum magnesium and depressive symptoms. Although depression is associated with inflammation, it is not primarily an inflammatory disease [47]. If an individual has a higher inflammation level that alters the pattern of brain signaling, then appropriate levels of magnesium function could be protective against depression. However, if one has a relatively mild inflammatory condition, then the effects of magnesium might be relatively low, which could lead to a null observation. In such a case, other alternative etiologies, such as psychosocial factors, which are not related to magnesium status, might represent more relevant risk factors.

Our analysis categorized inflammation caused by CRP into levels of 5 mg/L and 3 mg/L, respectively. For every 1 mg/dL increase in serum magnesium, depression score reduction was greater in the subgroup with CRP levels ≥ 5 mg/L (0.56 in the crude model, 0.54 in Model 1, 0.50 in Model 2) than in the subgroup with CRP levels ≥ 3 mg/L (0.46 in the crude model, 0.41 in Model 1, 0.38 in Model 2) (Figure 1). Those individuals with higher serum magnesium showed lower ORs of depression in the subgroup with CRP levels ≥ 5 mg/L (0.14 in the crude model, 0.11 in Model 1, 0.11 in Model 2) than in the subgroup with CRP levels ≥ 3 mg/L (0.21 in the crude model, 0.21 in Model 1, 0.23 in Model 2) (Figure 2). For participants taking serum magnesium as an antidepressant agent, the differences in effects with different thresholds shown in our results were compatible with a previous study [39]. Raison et al. examined the antidepressant effects of Infliximab, a tumor necrosis factor antagonist, in an intervention study. Only in subjects with baseline CRP levels above 5 mg/L did Infliximab show antidepressant effects and not in those with baseline CRP levels above 3 mg/L. Our results, together with the previous study, indicate that a subject’s baseline inflammation must be taken into consideration when assessing antidepressant effects and when dealing with certain agents.

CRP levels ranging from 3 to 10 mg/L are induced by chronic diseases, such as metabolic syndrome, and higher CRP levels have been associated with physical activity and other lifestyle factors, such as smoking [48]. In our study, we adjusted for chronic diseases, including diabetes mellitus, hypertension, and kidney diseases; for lifestyle covariates, including smoking, alcohol use, and physical activity status; and for covariates for socioeconomic status. However, we could not exclude all possible residual confounding factors. Additionally, we did not assess acute infections while drawing blood, which can affect CRP levels considerably. However, the study participants were community-dwelling adults who voluntarily participated in the study, and thus had low likelihoods of harboring major acute infections at the time of the study. Our analysis shows that the effects of CRP on depression remain significant after controlling for serum magnesium, and the effects of serum magnesium diminished after controlling for CRP (Table 2). Therefore, partial mediation effects of CRP on the association between serum magnesium and depressive symptoms would be probable. A similar scenario also was observed in a study assessing the effects of depressive symptoms on atherosclerosis. The effects of depressive symptoms diminished after controlling for IL-6 and CRP [49].

This study has some strengths. First, we analyzed the data of national representative samples of Taiwanese community-dwelling adults. Second, we included potential confounding factors related to depression. Third, this study reported the association between serum magnesium levels and depression scores after stratification by gender and different inflammation levels. In addition, we conducted a dose–response analysis to better demonstrate the association of serum magnesium with depression.

Potential limitations of this study are as follows. First, serum magnesium levels have been reported to vary over phases of the menstrual cycle. Therefore, the measurements of serum magnesium levels among female participants might have been biased and led to null results. Second, the CRP might be affected by acute infection/chronic illness or medication, which could not be accounted for in the analysis. Third, depressive symptoms were self-reported. Fourth, the study participants were healthy, community-dwelling subjects, who might exhibit different characteristics than patients with major depressive disorder. The relationship between serum magnesium, CRP, and depression in subjects with major depressive disorder needs further examination. Fifth, this is an association study that hinders further inference of causality. The mechanism underlying the relationship between serum magnesium levels, inflammation levels, and the development of depression is unclear, and understanding the role of inflammation in modulating the protective effects of magnesium against depression requires further exploration. The study results might also support further intervention studies for investigating the protective effects of serum magnesium against depression in healthy populations exhibiting different inflammation levels.

## 5. Conclusions

In conclusion, a dose–response relationship was observed between higher serum magnesium levels and a lower depression risk, and this relationship was affected by CRP levels. Further prospective studies are necessary to determine the causality between magnesium status and depression risk, and the complexity of the role of inflammation.

## Figures and Tables

**Table 1:** Characteristics of predictors by depression score as a continuous or binary variable.

Variable	Total Sample (*n* = 2196)	Depression (Continuous Variable) ^1^		Depression (Binary Variable) ^2^		
		Mean ± SD	Median (IQR)	Depression < 2, Mean ± SD	Depression ≥ 2, Mean ± SD	*p* Value ^3^
**Serum Mg (mg/dL)**		2.14 ± 0.19	2.14 (0.27)	2.14 ± 0.19	2.10 ± 0.21	0.018 *
**Age**		53.39 ± 17.36	54.00 (27.00)	53.75 ± 17.41	48.30 ± 15.85	<0.001 *
	***n* (%)**	**Mean ± SD**	***p* Value ^3^**	**Depression < 2,** ***n* (%)**	**Depression ≥ 2,** ***n* (%)**	
**CRP**						
<5 mg/L	1976 (89.98)	0.34 ± 0.67	0.092	1855 (90.44)	121 (83.45)	0.007 *
≥5 mg/L	220 (10.02)	0.44 ± 0.86		196 (9.56)	24 (16.55)	
**Sex**						
Women	1127 (51.32)	0.38 ± 0.70	0.023 *	1050 (51.19)	77 (53.10)	0.647
Men	1069 (48.68)	0.32 ± 0.69		1001 (48.81)	68 (46.90)	
**BMI**			0.003 *			0.043 *
<18	118 (5.37)	0.42 ± 0.66		111 (5.42)	7 (4.83)	
18–24	933 (42.49)	0.40 ± 0.76		857 (41.78)	76 (52.41)	
>24	1145 (52.14)	0.30 ± 0.63		1083 (52.80)	62 (42.76)	
**Education level**						
None	209 (9.52)	0.37 ± 0.72	0.457	194 (9.46)	15 (10.34)	0.248
Below SHS	936 (42.62)	0.33 ± 0.67		885 (43.15)	51 (35.17)	
SHS	585 (26.64)	0.38 ± 0.72		538 (26.23)	47 (32.41)	
Above SHS	466 (21.22)	0.35 ± 0.71		434 (21.16)	32 (22.07)	
**Income level**						
No income	533 (24.27)	0.40 ± 0.75	0.055	489 (23.84)	44 (30.34)	0.011 *
TWD 20,000 and below	842 (38.34)	0.31 ± 0.64		803 (39.15)	39 (26.90)	
TWD 20,001–50,000	569 (25.91)	0.38 ± 0.71		525 (25.60)	44 (30.34)	
TWD 50,001–70,000	153 (6.97)	0.25 ± 0.65		146 (7.12)	7 (4.83)	
TWD 70,001 and above	99 (4.51)	0.38 ± 0.74		88 (4.29)	11 (7.59)	
**Physical activity status**						
No	978 (44.55)	0.40 ± 0.76	0.010 *	898 (43.78)	80 (55.17)	0.028 *
Yes–walking	615 (27.95)	0.30 ± 0.61		583 (28.43)	32 (22.07)	
Yes–more than walking ^4^	603 (27.50)	0.32 ± 0.66		570 (27.79)	33 (22.76)	
**Alcohol use**						
Non-drinker	1186 (54.01)	0.34 ± 0.67	0.198	1111 (54.17)	75 (51.72)	0.073
Former drinker	178 (8.11)	0.44 ± 0.85		159 (7.75)	19 (13.10)	
Current drinker	832 (37.89)	0.35 ± 0.69		781 (38.08)	51 (35.17)	
**Smoking Status**						
Non-smoker	1493 (67.99)	0.34 ± 0.65	0.223	1407 (68.60)	86 (59.31)	0.034 *
Former smoker	272 (12.39)	0.33 ± 0.70		253 (12.34)	19 (13.10)	
Current smoker	431 (19.63)	0.40 ± 0.83		391 (19.06)	40 (27.59)	
**Diabetes mellitus**						
No	2007 (91.39)	0.35 ± 0.69	0.818	1874 (91.37)	133 (91.72)	0.883
Yes	189 (8.61)	0.34 ± 0.69		177 (8.63)	12 (8.28)	
**Hypertension**						
No	1665 (75.82)	0.36 ± 0.72	0.088	1550 (75.57)	115 (79.31)	0.310
Yes	531 (24.18)	0.31 ± 0.60		501 (24.43)	30 (20.69)	
**Kidney disease**						
No	2156 (98.18)	0.35 ± 0.69	0.279	2017 (98.34)	139 (95.86)	0.031 *
Yes	40 (1.82)	0.50 ± 0.88		34 (1.66)	6 (4.14)	

CRP: C-reactive protein; SD: standard deviation; SE: standard error; SHS: senior high school; Mg: magnesium; * *p* < 0.05. ^1^ Depression score ranges from 0 to 4. ^2^ Depression status was categorized into two groups based on the scores on BSRS-5: (1) depression score < 2 and (2) depression score ≥ 2. ^3^ Results of Fisher’s exact test and the chi-square test for categorical variables, the *t* test or ANOVA test for continuous variables, and Pearson correlation coefficient for continuous variables. ^4^ Under the more than walking status, physical activities included running, hiking, folk dancing, aerobic dancing, swimming, and bicycling.

**Table 2:** Odds ratios and estimates of depression scores according to serum Mg and CRP levels, NAHSIT 2005–2008.

Variable	Depression (Continuous Variable) ^1^			Depression (Binary Variable) ^2^		
	β	*p* Value	95% CI	OR	*p* Value	95% CI
*Crude*						
Serum Mg (mg/dL)	−0.26	0.001 *	(−0.41, −0.11)	0.32	0.008 *	(0.14, 0.74)
CRP ≥ 5 mg/L ^3^	0.10	0.040	(0.01, 0.20)	1.88	0.008 *	(1.18, 2.98)
*Model 1*						
Serum Mg (mg/dL)	−0.23	0.004 *	(−0.39, −0.08)	0.36	0.018 *	(0.15, 0.84)
CRP ≥ 5 mg/L	0.14	0.005 *	(0.04, 0.24)	2.23	0.001 *	(1.37, 3.61)
*Model 2*						
Serum Mg (mg/dL)	−0.23	0.004 *	(−0.38, −0.07)	0.37	0.022 *	(0.16, 0.87)
CRP ≥ 5 mg/L	0.14	0.005 *	(0.04, 0.24)	2.19	0.002 *	(1.35, 3.55)

CI: confidence interval; CRP: C-reactive protein; Mg: magnesium; * *p* < 0.05. ^1^ Depression scores range from 0 to 4. ^2^ Depression status was categorized into two groups based on the scores from the BSRS-5: (1) depression scores of <2 or (2) depression scores of ≥2. ^3^ Reference group was CRP levels < 5 mg/L. Model 1 was adjusted for age, sex, and BMI. Model 2 was adjusted for statistically significant variables (*p* < 0.05) in the univariate analysis (Table 1).

**Table 3:** Odds ratios and estimates of depression scores according to serum Mg levels by CRP levels, NAHSIT 2005–2008.

Variable	Depression (Continuous Variable) ^1^			Depression (Binary Variable) ^2^		
	β	*p* Value	95% CI	OR	*p* Value	95% CI
Subgroup analysis (among CRP ≥ 5 mg/L)						
*Crude*						
Serum Mg (mg/dL)	−0.56	0.019 *	(−1.02, −0.09)	0.14	0.018 *	(0.03, 0.72)
*Model 1*						
Serum Mg (mg/dL)	−0.54	0.025 *	(−1.01, −0.07)	0.11	0.013 *	(0.02, 0.62)
*Model 2*						
Serum Mg (mg/dL)	−0.50	0.039 *	(−0.97, −0.03)	0.11	0.018 *	(0.02, 0.69)
**Subgroup analysis (among CRP < 5 mg/L)**						
*Crude*						
Serum Mg (mg/dL)	−0.19	0.025 *	(−0.35, −0.02)	0.49	0.159	(0.18, 1.32)
*Model 1*						
Serum Mg (mg/dL)	−0.17	0.042 *	(−0.34, −0.01)	0.49	0.170	(0.18, 1.35)
*Model 2*						
Serum Mg (mg/dL)	−0.17	0.045 *	(−0.33, −0.00)	0.51	0.186	(0.19, 1.39)

CI: confidence interval; CRP: C-reactive protein; Mg: magnesium; * *p* < 0.05. ^1^ Depression scores range from 0 to 4. ^2^ Depression status was categorized into two groups based on the scores from BSRS-5: (1) depression scores of <2 and (2) depression scores of ≥2. Model 1 was adjusted for age, sex, and BMI. Model 2 was adjusted for statistically significant variables (*p* < 0.05) in the univariate analysis (Table 1).

## Data Availability

Not applicable.

## Supplementary Materials

The following supporting information can be downloaded at: https://www.mdpi.com/article/10.3390/nu15071560/s1, Table S1: Characteristics of included and excluded samples. Table S2. Odds ratios and estimates of depression according to serum Mg and CRP levels, NAHSIT 2005–2008. Table S3. Odds ratios and estimates of depression according to serum Mg levels by CRP levels, NAHSIT 2005–2008. Table S4. Odds ratios and estimates of depression according to serum Mg and CRP levels by sex, NAHSIT 2005–2008. Table S5. Odds ratios and estimates of depression according to serum Mg and CRP levels by sex, NAHSIT 2005–2008.
