# Supplementary material for: Elevated C-Reactive Protein Levels Modify the Effect of Magnesium on Depressive Symptoms: A Population-Based Study
Source: Nutrients. 2023 Mar 23;15(7):1560. doi: 10.3390/nu15071560 (PMC10097277; doi:10.3390/nu15071560)
Supplement: Supplementary file 1 [file nutrients-15-01560-s001.zip › nutrients-2277392-supplementary.pdf]

nutrients MDPI

Table S1. Characteristics of included and excluded samples

Variable	Total sample N (%)	Included sample Mean ± SD	Excluded sample Mean ± SD	Standardized mean difference[1]
**Depression**	4,210 (91.22)	0.35 ± 0.69	0.36 ± 0.68	0.02
**Serum Mg (mg/dL)**	2,649 (57.40)	2.14 ± 0.19	2.15 ± 0.20	0.07
**Age**	4,615 (100.00)	53.39 ± 17.36	52.62 ± 18.64	−0.04
		**n (%)**	**n (%)**	
**Depression**				
<2	3,925 (93.23)	2,051 (93.40)	1,874 (93.05)	0.01
≥2	285 (6.77)	145 (6.60)	140 (6.95)	
**CRP (mg/L)**				
<5	2,405 (89.94)	1,976 (89.98)	429 (89.75)	0.03
≥5	269 (10.06)	220 (10.02)	49 (10.25)	
**Sex**				
Women	2,322 (50.31)	1,127 (51.32)	1,195 (49.40)	0.04
Men	2,293 (49.69)	1,069 (48.68)	1,224 (50.60)	
**Education level**				
None	491 (10.66)	209 (9.52)	282 (11.71)	0.16
Below SHS	2,082 (45.21)	936 (42.62)	1,146 (47.57)	
SHS	1,190 (25.84)	585 (26.64)	605 (25.11)	
Above SHS	842 (18.28)	466 (21.22)	376 (15.61)	
**Income level**				
No income	1,040 (23.89)	533 (24.27)	507 (23.49)	0.14
NT$20,000 and below	1,774 (40.74)	842 (38.34)	932 (43.19)	
NT$20,001–50,000	1,101 (25.29)	569 (25.91)	532 (24.65)	
NT$50,001–70,000	279 (6.41)	153 (6.97)	126 (5.84)	
NT$70,001 and above	160 (3.67)	99 (4.51)	61 (2.83)	
**Physical activity status**				
No	1,909 (47.11)	978 (44.54)	931 (50.16)	0.12
Yes–walking	1,139 (28.11)	615 (28.01)	524 (28.23)	
Yes–more than walking[2]	1,004 (24.78)	603 (27.46)	401 (21.61)	
**Alcohol use**				
Non-drinker	2,277 (53.54)	1,186 (54.01)	1,091 (53.04)	0.02
Former drinker	348 (8.18)	178 (8.11)	170 (8.26)	
Current drinker	1,628 (38.28)	832 (37.89)	796 (38.70)	
**Smoking status**				
Non-smoker	2,674 (64.53)	1,493 (67.99)	1,181 (60.63)	0.17
Former smoker	501 (12.09)	272 (12.39)	229 (11.76)	
Current smoker	969 (23.38)	431 (19.63)	538 (27.62)	
**Diabetes mellitus**				
No	3,971 (90.77)	2,007 (91.39)	1,964 (90.13)	0.04
Yes	404 (9.23)	189 (8.61)	215 (9.87)	
**Hypertension**				
No	3,311 (75.75)	1,665 (75.82)	1,646 (75.68)	0.00
Yes	1,060 (24.25)	531 (24.18)	529 (24.32)	
**Kidney disease**				
No	4,275 (97.96)	2,156 (98.18)	2,119 (97.74)	0.03
Yes	89 (2.04)	40 (1.82)	49 (2.26)	

CRP: C-reactive protein; SD: standard deviation; SE: standard error; SHS: senior high school; Mg: magnesium; *$p < 0.05$.

[1] Standardized difference = difference in means or proportions divided by the standard error.

[2] Physical activities considered as more than walking included running, hiking, folk dancing, aerobic dancing, swimming, and bicycling.

Table S2. Odds ratios and estimates of depression according to serum Mg and CRP levels, NAHSIT 2005–2008

	Depression (continuous variable)[1]			Depression (binary variable)[2]		
**Variable**	**β**	**p value**	**95% CI**	**OR**	**p value**	**95% CI**
Crude						
Serum Mg (mg/dL)	−0.26	0.001*	(−0.41, −0.11)	0.32	0.008*	(0.14, 0.74)
CRP ≥ 3 mg/L[3]	0.04	0.249	(−0.03, 0.12)	1.54	0.033*	(1.04, 2.28)
Model 1						
Serum Mg (mg/dL)	−0.24	0.003*	(−0.39, −0.08)	0.35	0.018*	(0.15, 0.83)
CRP ≥ 3 mg/L	0.09	0.018*	(0.02, 0.17)	1.93	0.002*	(1.17, 2.94)
Model 2						
Serum Mg (mg/dL)	−0.23	0.004*	(−0.39, −0.07)	0.37	0.021*	(0.16, 0.86)
CRP ≥ 3 mg/L	0.09	0.019*	(0.02, 0.17)	1.91	0.003*	(1.26, 2.90)

CI: confidence interval; CRP: C-reactive protein; Mg: magnesium; *$p < 0.05$.

[1] Depression score ranges from 0 to 4.

[2] Depression status was categorized into two groups based on the scores on BSRS-5: (1) depression score < 2, and (2) depression score ≥ 2.

[3] Reference group was CRP < 3 mg/L.

Model 1 adjusted for age, sex and BMI.

Model 2 adjusted for statistically significant variables ($p < 0.05$) in the univariate analysis (Table 1).

Table S3. Odds ratios and estimates of depression according to serum Mg levels by CRP levels, NAHSIT 2005–2008

	Depression (continuous variable)[1]			Depression (binary variable)[2]		
**Variable**	**β**	**p value**	**95% CI**	**OR**	**p value**	**95% CI**
**Subgroup analysis (among CRP ≥ 3 mg/L)**						
Crude						
Serum Mg (mg/dL)	−0.46	0.008*	(−0.79, −0.12)	0.21	0.026*	(0.05, 0.83)
Model 1						
Serum Mg (mg/dL)	−0.41	0.022*	(−0.75, −0.06)	0.21	0.033*	(0.05, 0.88)
Model 2						
Serum Mg (mg/dL)	−0.38	0.037*	(−0.73, −0.03)	0.23	0.047*	(0.05, 0.98)
**Subgroup analysis (among CRP < 3 mg/L)**						
Crude						
Serum Mg (mg/dL)	−0.18	0.041*	(−0.36, −0.00)	0.46	0.149	(0.16, 1.32)
Model 1						
Serum Mg (mg/dL)	−0.16	0.066	(−0.34, 0.01)	0.45	0.148	(0.16, 1.33)
Model 2						
Serum Mg (mg/dL)	−0.16	0.071	(−0.34, 0.01)	0.46	0.154	(0.15, 1.34)

CI: confidence interval; CRP: C-reactive protein; Mg: magnesium; *$p < 0.05$.

1 Depression score ranges from 0 to 4.

2 Depression status was categorized into two groups based on the scores on BSRS-5: (1) depression score < 2 and (2) depression score ≥ 2.

Model 1 adjusted for age, sex and BMI.

Model 2 adjusted for statistically significant variables ($p < 0.05$) in the univariate analysis (Table 1).

Table S4. Odds ratios and estimates of depression according to serum Mg and CRP levels by sex, NAHSIT 2005–2008

	Depression (continuous variable)[1]			Depression (binary variable)[2]		
**Variable**	**β**	**p value**	**95% CI**	**OR**	**p value**	**95% CI**
**Subgroup analysis (among men)**						
Crude						
Serum Mg (mg/dL)	−0.37	<0.001*	(−0.59, −0.16)	0.24	0.016*	(0.08, 0.77)
CRP ≥ 5 mg/L[3]	0.09	0.205	(−0.05, 0.22)	1.45	0.293	(0.73, 2.91)
Model 1						
Serum Mg (mg/dL)	−0.37	<0.001*	(−0.58, −0.15)	0.25	0.020*	(0.08, 0.81)
CRP ≥ 5 mg/L	0.12	0.087	(−0.02, 0.25)	1.64	0.133	(0.81, 3.32)
Model 2						
Serum Mg (mg/dL)	−0.38	<0.001*	(−0.59 −0.16)	0.25	0.019*	(0.08, 0.80)
CRP ≥ 5 mg/L	0.12	0.074	(-0.01, 0.26)	1.75	0.126	(0.85, 3.57)
**Subgroup analysis (among women)**						
Crude						
Serum Mg (mg/dL)	−0.12	0.300	(−0.34, 0.10)	0.51	0.281	(0.15, 1.73)
CRP ≥ 5 mg/L	0.09	0.200	(−0.05, 0.23)	2.10	0.022*	(1.11, 3.95)
Model 1						
Serum Mg (mg/dL)	−0.09	0.418	(−0.32, 0.13)	0.53	0.323	(0.15 1.88)
CRP ≥ 5 mg/L	0.16	0.030*	(0.02, 0.31)	2.58	0.006*	(1.32, 5.05)
Model 2						
Serum Mg (mg/dL)	−0.07	0.550	(−0.29, 0.16)	0.59	0.421	(0.16, 2.14)
CRP ≥ 5 mg/L	0.15	0.050*	(-0.00, 0.29)	2.42	0.010*	(1.23, 4.74)

CI: confidence interval; CRP: C-reactive protein; Mg: magnesium; OR: odds ratio; *$p < 0.05$.
[1] Depression score ranges from 0 to 4.
[2] Depression status was categorized into two groups based on the scores on BSRS-5: (1) depression score < 2 and (2) depression score ≥ 2.
[3] Reference group was CRP < 5 mg/L.
Model 1 adjusted for age, and BMI.
Model 2 adjusted for statistically significant variables ($p < 0.05$) in univariate analysis (Table 1).

Table S5. Odds ratios and estimates of depression according to serum Mg and CRP levels by sex, NAHSIT 2005–2008

	Depression (continuous variable)[1]			Depression (binary variable)[2]		
**Variable**	**β**	**p value**	**95% CI**	**OR**	**p value**	**95% CI**
**Subgroup analysis (among men)**						
Crude						
Serum Mg (mg/dL)	−0.37	<0.001*	(−0.60, −0.16)	0.23	0.009*	(0.07, 0.74)
CRP ≥ 3 mg/L[3]	0.06	0.255	(−0.04, 0.17)	1.21	0.304	(0.67, 2.20)
Model 1						
Serum Mg (mg/dL)	−0.36	0.001*	(−0.58, −0.15)	0.25	0.019*	(0.08, 0.80)
CRP ≥ 3 mg/L	0.11	0.056	(−0.00, 0.21)	1.50	0.196	(0.81, 2.80)
Model 2						
Serum Mg (mg/dL)	−0.37	<0.001*	(−0.59 −0.16)	0.25	0.01*	(0.08, 0.79)
CRP ≥ 3 mg/L	0.11	0.046*	(0.00, 0.22)	1.52	0.186	(0.82, 2.83)
**Subgroup analysis (among women)**						
Crude						
Serum Mg (mg/dL)	−0.12	0.290	(−0.34, 0.10)	0.50	0.268	(0.15, 1.70)
CRP ≥ 3 mg/L	0.01	0.892	(−0.10, 0.11)	1.71	0.049*	(1.00, 2.92)
Model 1						
Serum Mg (mg/dL)	−0.10	0.396	(−0.32, 0.13)	0.51	0.304	(0.14 1.83)
CRP ≥ 3 mg/L	0.08	0.194	(−0.01, 0.19)	2.23	0.008*	(1.24, 4.02)
Model 2						
Serum Mg (mg/dL)	−0.07	0.528	(−0.30, 0.15)	0.58	0.401	(0.16, 2.10)
CRP ≥ 3 mg/L	0.07	0.246	(-0.05, 0.18)	2.16	0.010*	(1.20, 3.89)

CI: confidence interval; CRP: C-reactive protein; Mg: magnesium; OR: odds ratio; *p < 0.05.
[1] Depression score ranges from 0 to 4.
[2] Depression status was categorized into two groups based on the scores on BSRS-5: (1) depression score < 2 and (2) depression score ≥ 2.
[3] Reference group was CRP < 3 mg/L.
Model 1 adjusted for age, and BMI.
Model 2 adjusted for statistically significant variables (p < 0.05) in univariate analysis (Table 1).
